# Estimating Zenith Tropospheric Delays from BeiDou Navigation Satellite System Observations

**DOI:** 10.3390/s130404514

**Published:** 2013-04-03

**Authors:** Aigong Xu, Zongqiu Xu, Maorong Ge, Xinchao Xu, Huizhong Zhu, Xin Sui

**Affiliations:** 1 School of Geomatics, Liaoning Technical University, No. 47 Zhonghua Road, Fuxin 123000, China; E-Mails: xu_ag@126.com (A.X.); zongqiuxu@gmail.com (Z.X.); xuxinchao84@163.com (X.X.); zhuhuizhong2002@163.com (H.Z.); survey_suixin@163.com (X.S.); 2 Department of Geodesy and Remote Sensing, German Research Center for Geosciences, Potsdam 14473, Germany

**Keywords:** BeiDou navigation satellite system, precise point positioning, network solution, GNSS meteorology

## Abstract

The GNSS derived Zenith Tropospheric Delay (ZTD) plays today a very critical role in meteorological study and weather forecasts, as ZTDs of thousands of GNSS stations are operationally assimilated into numerical weather prediction models. Recently, the Chinese BeiDou Navigation Satellite System (BDS) was officially announced to provide operational services around China and its neighborhood and it was demonstrated to be very promising for precise navigation and positioning. In this contribution, we concentrate on estimating ZTD using BDS observations to assess its capacity for troposphere remote sensing. A local network which is about 250 km from Beijing and comprised of six stations equipped with GPS- and BDS-capable receivers is utilized. Data from 5 to 8 November 2012 collected on the network is processed in network mode using precise orbits and in Precise Point Positioning mode using precise orbits and clocks. The precise orbits and clocks are generated from a tracking network with most of the stations in China and several stations around the world. The derived ZTDs are compared with that estimated from GPS data using the final products of the International GNSS Service (IGS). The comparison shows that the bias and the standard deviation of the ZTD differences are about 2 mm and 5 mm, respectively, which are very close to the differences of GPS ZTD estimated using different software packages.

## 1. Introduction

For more than one decade China has been working on its own BeiDou navigation satellite system (abbreviated to BDS) [1]. On 27 December 2012, the regional system was officially announced to provide operational positioning services over the Asia-Pacific region. Due to its similar signal structure and analogous frequencies with respect to that of the American GPS, BDS-capable multi-GNSS receivers had been developed before its Interface Control Document (ICD) [2] was publically disclosed. These receivers are utilized in a number of studies on BDS precise orbit determination and clock estimation [3–8], Precise Point Positioning (PPP) [9] and relative positioning [3,7,10]. All the studies confirm that BDS is able to provide positioning and navigation services of similar accuracy to GPS.

Besides precise positioning, GNSS is also employed to retrieve tropospheric information, such as zenith tropospheric delays (ZTD) or slant tropospheric delays, for meteorological study and weather forecasts [11]. The GPS derived ZTD was validated of an accuracy of about 6 to 10 mm by various technologies or instruments, such as radiosonde, water vapor radiometer, and Very Long Baseline Interferometry (VLBI) [12–14]. Today GNSS-derived ZTD plays a very important role in operational weather forecasts, as ZTD from thousands of GNSS stations are assimilated into numerical weather models in near real-time [15,16]. However, scientists have proved that more stations and more satellites are needed for models of higher spatial and temporal resolution [17]. Therefore, BDS consisting five Geostationary Earth Orbit (GEO), five Inclined Geosynchronous Orbit (IGSO) and four Medium Earth Orbit (MEO) satellites now and another 23 additional MEO satellites expected by 2020 [1,2] will provide a huge number of observations and ensure significant improvements to current GNSS meteorology. Thus, in this contribution we concentrate on retrieving tropospheric delays from BDS observations.

There are two data processing modes for estimating ZTD from GNSS observations: PPP mode and network mode. In the PPP mode, precise satellite orbits and clocks must be available and are fixed as known in the processing, so that data can be processed station-by-station. This strategy is very computationally efficient and can be performed for any number of stations. In the network mode, a number of stations are processed together where satellite clocks are estimated as additional parameters or cancelled by forming differential observations between stations. Although the network mode is really time-consuming compared to the PPP mode, precise satellite clocks are not required as a pre-condition. It is already confirmed with a large set of GPS data that the two processing schemes provide ZTD results of similar quality [12].

In this study, a test network comprising six stations equipped with GPS- and BDS-capable dual-frequency receivers is deployed in Hebei Province with an inter-station distance of about 100 km. GPS and BDS data from this network are processed independently in both network and PPP mode to estimate ZTDs. The BDS-derived estimates are validated by comparing with that of GPS. The assessment shows that the bias and standard deviation (STD) of the ZTD differences are 2 mm and 5 to 6 mm, respectively, which is similar to the differences of GPS ZTD derived from different software packages [12].

## 2. Tracking Networks

In order to carry out PPP for BDS observations of a local or regional network, precise orbit and clock products must be computed in advance. By the way, for network solutions, precise orbits are also needed to get rid of the broadcast orbit errors. Therefore, a global network is required for precise orbit determination and clock estimation.

**Figure 1.**
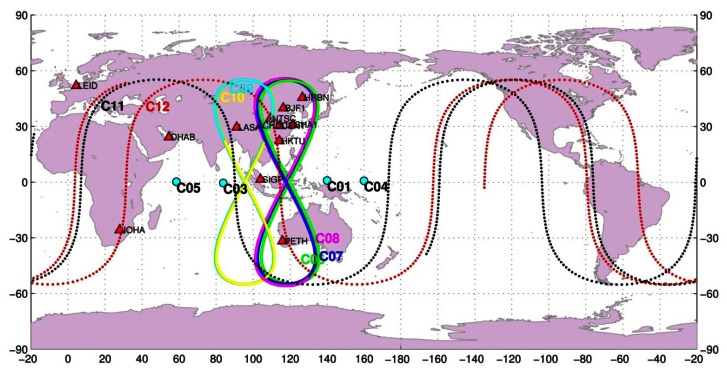
Ground tracks of BDS GEO satellites (C01, C03, C04, C05), IGSO satellites (C06, C07, C08, C09, G10), and MEOs (C11, C12) and station distribution of the BETS experimental tracking stations [8].

The BeiDou Experimental Test Service (BETS) network with BDS and GPS capacity has been deployed for scientific purposes by the GNSS research center at Wuhan University and is now extending to a global tracking network. Since March 2011, 14 stations have already been deployed in China and its neighboring regions. Among these, nine stations are located inside the territory of China and five overseas. The stations in China are BJF1 in Beijing, CENT in Wuhan, CHDU in Chengdu, HRBN in Harbin, HKTU in Hong Kong, NTSC and XIAN in Xi'an city, SHAO in Shanghai, and LASA in Tibet. The five overseas stations are SIGP (Singapore), PETH (Australia), DHAB (the United Arab Emirates), LEID (The Netherlands), and JOHA (South Africa). The station distribution is shown in Figure 1. All the stations are equipped with the UR240 dual-frequency and GPS/BDS dual-system receivers and the UA240 antennas manufactured by the Unicore Company (Beijing, China; http://www.unicorecomm.com/english/). A number of studies were carried out based on data of this network [5–8]. The precise orbits and clocks of the BDS satellites used in this study are computed from this network using the strategy by He et al. [8]. Furthermore, several BETS stations are involved into the network solution for reducing the spatial correlation of ZTD parameters to obtain so called absolute ZTDs [11,18].

**Figure 2.**
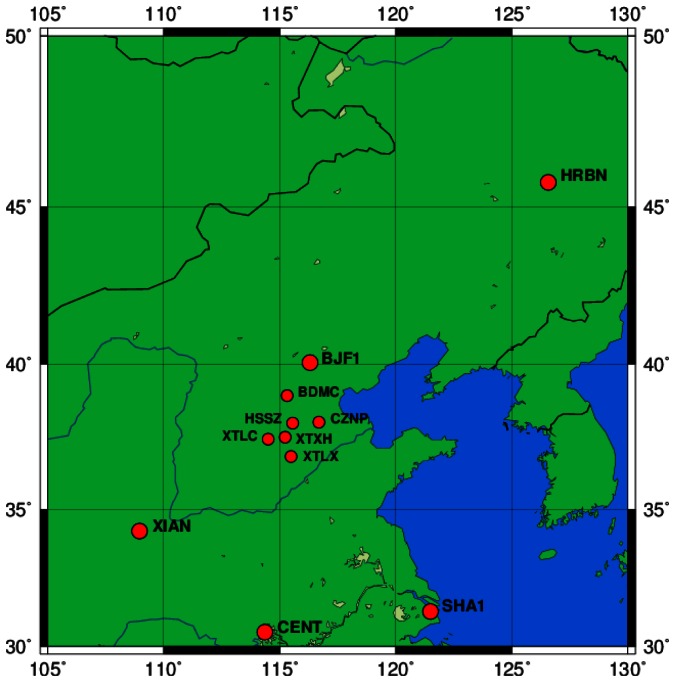
The local GNSS network of medium inter-station distance equipped with GPS- and BDS-capable dual-frequency receivers for ZTD estimation.

A local network with six stations equipped with the same Unicore receiver and antenna are employed as sensor stations for estimating ZTDs. The network is about 250 km from Beijing and is deployed for Network Real-Time Kinematic (NRTK) services with GPS and planned to be extended for GPS and BDS multi-GNSS service, so the inter-station distance is about 100 km on average. The local network is shown in Figure 2 with the BETS stations BJF1 (Beijing) and Harbin (HRBN) in its northern and XIAN, CENT (Wuhan) and SHA1 (Shanghai) in its southern part.

Data from 5 November 2012 (day of year 310) to 8 November 2012 (day of year 314) of the local network is provided by the Chinese Academy of Survey and Mapping for this study. In order to obtain precise orbits for network solution and precise orbits and clocks for PPP, data from 309 (November 4) to 315 (November 9) in 2012 of the BETS are used to generate the products using the three-day solution strategy described by He *et al.* [8].

During the selected period, four GEO, five IGSO and two MEO satellites were in operation. One GEO and two other MEO satellites were still in test phase and could not be involved in the processing. The ground tracks of the satellites are also shown in Figure 1.

## 3. Data Processing

The Positioning And Navigation Data Analyst (PANDA) software was used for the data processing. The software was developed at the GNSS Research Center in Wuhan University several years ago [19,20] and was adapted recently for BDS data processing [5,6]. Similar to most scientific GNSS software packages, it includes the following basic modules: data preprocessing, orbit integration, parameter estimation, data editing and ambiguity-fixing. Two estimators are developed: a least squares estimator for post-mission processing and a square root information filter estimator for real-time processing.

The software package can be configured via a control file to run for various applications, such as (near real-time) orbit determination, (real-time) satellite clock estimation, and PPP with precise orbit and clock products.

As BDS is very similar in signal structure and frequencies to GPS, the observation models and satellite force models for GPS can be utilized directly for BDS with very slight modifications. Therefore, observable models and processing parameters are similar to the operational ZTD estimation at the German Research Center for Geosciences (GFZ) listed in Table 1.

It should be mentioned that Phase Center Offset (PCO) and Phase Center Variation (PCV) of neither satellites nor receivers are available now. Satellite attitude control mode is also not yet announced. The differences in PCO and PCV could cause significant biases in ZTD estimates [21]. Usually the PCV file generated and released by the International GNSS Service (IGS) [22] which includes corrections for both satellites and receivers should be used for precise data processing [23]. Unfortunately, until now there is no information available for BDS satellites and the Unicore antenna. Therefore, PCO and PCV corrections are neglected. As the receivers of the same type are equipped in the experiment networks, this neglect may not cause serious problem. Moreover, the attitude control mode of the BDS satellites is assumed to be the same as that of the GPS Block IIR satellites.

In both PPP and network processing mode, ionosphere-free phase and range observations are utilized with proper weights in order to eliminate the ionospheric delays. We process every 24-hour data in one batch, so that the estimates are strong enough. Tropospheric delays are first corrected through ZTD computed with Saastamoinen model using meteorological data from the Global Pressure and Temperature model (GPT) [24] and the Vienna Mapping Function (VMF) [25]. The remaining ZTD are modeled by a random-walk stochastic process with a power density of $2.5\text{mm}/\sqrt{\text{h}}$ [26] and ZTD horizontal gradients are not considered.

Satellite orbits and station coordinates are fixed or tightly constrained to their precisely estimated values. Receiver clocks are estimated as white noise epoch-by-epoch. Ambiguities are estimated as real-values and ambiguity-fixing is not undertaken because it brings almost no improvement on ZTD if the session is long enough [27]. Furthermore, ambiguity-fixing over long baselines is not such reliable for BDS because its range quality is not good enough [8]. In the case of network solution, satellite clocks must be estimated epoch-by-epoch, whereas they are fixed as known in PPP mode. Be aware that the local stations are not included in the estimation of the precise orbits and clocks used here.

In GPS data processing for meteorological applications, when a network solution is performed at regional scale, a minimal geographical extension of 500 to 2,000 km is necessary in order to reduce the correlation between ZTD estimates at the different stations to obtain absolute ZTD parameters [11,18]. Therefore, several BETS stations which are far away from the local network must be included into the network solution.

The same PPP and network processing procedures are also applied to the GPS data of the same stations but with IGS final orbits and clocks to provide a reference for comparison. Therefore we have carried out four processing scenarios: PPP and network solution using BDS data referred as to BDSPPP and BDSNET, respectively; and PPP and network solution using GPS data, referred as to GPSPPP and GPSNET. For the network solutions, CENT, SHA1 and HRBN from the BETS network are processed together with the six local stations.

## 4. Results

As results, the number of observations and the residuals of the observations are first presented and compared with that of GPS. Then the ZTD derived from BDS and GPS in PPP and network mode are compared and assessed.

### 4.1. Observations

**Figure 3.**
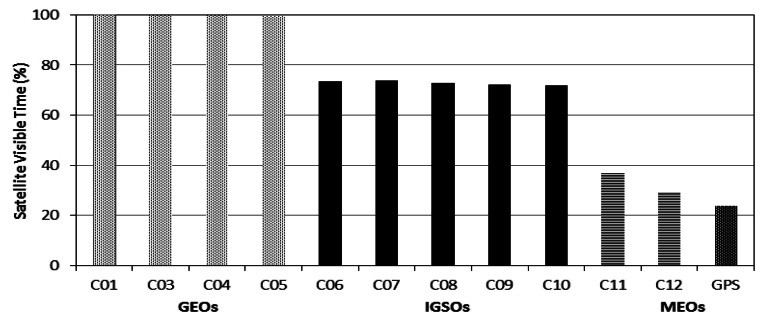
Satellite visibilities in percentage of time for the local network. The GEOs are observed all the time, for IGSO and MEO the averaged visible windows are 73% and 33%, respectively. For GPS satellites the averaged visible time is about 25%.

The special constellation of the BDS regional system leads to a very different satellite visibility from the GPS system, as most of the satellites are GEOs and IGSOs. Figure 3 shows the percentage of time each satellite can be observed by the local network. The GEO satellites are observed all the time, as their movement in the sky is very slight. The IGSO satellites can be observed 73% over time, because they are limited in a narrow longitude zone. For BDS MEOs, the averaged visible window is about 33%, larger than that for GPS satellites of about 25%. Obviously, most of the transmitting paths do not change (GEO), or do not change very much (IGSO). Therefore, satellite distribution in sky might have a very strong impact on ZTD estimation. In general, satellites should be distributed zenith-symmetrically and evenly for retrieving the most precise ZTDs.

**Figure 4.**
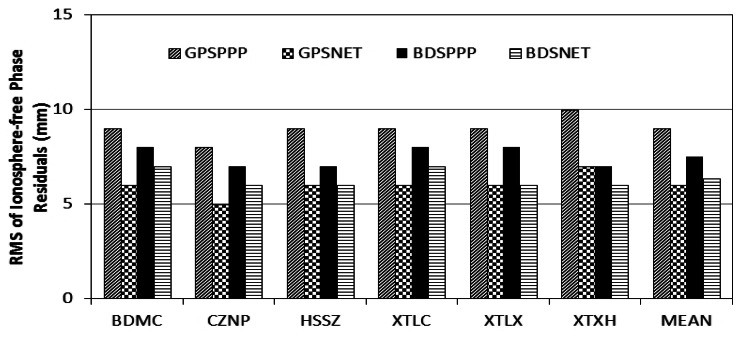
RMS of the ionosphere-free phase observations of the four processing scenarios. RMS of PPP solution is slightly larger than that of the related network solution. GPS and BDS have similar phase noise.

Figure 4 shows the root mean square (RMS) errors of the ionosphere-free phase observations of the four processing scenarios. In general, the RMS of PPP solution is slightly larger than that of the network solutions. The GPSPPP solution has the largest RMS because the software package and processing procedure are not fully consistent with that for generating the IGS final products. Anyway, the differences are rather small of about 1 to 2 mm and confirm that BDS and GPS have similar phase noises.

**Figure 5.**
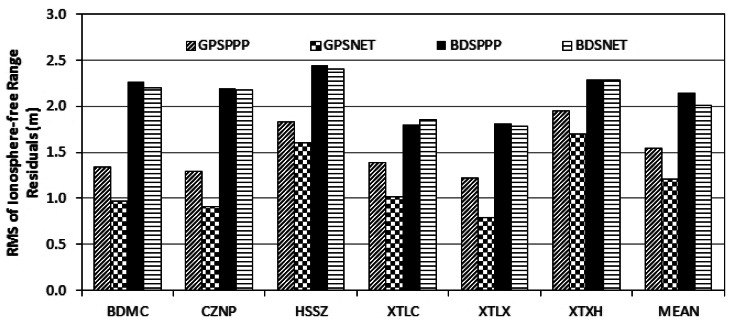
RMS of the ionosphere-free range observations of the four processing scenarios. Both PPP and network solutions have similar range RMS. BDS range RMS is twice as large as that of GPS.

Figure 5 shows the RMS of the ionosphere-free range observations. If receivers of the same type are used, the Differential Code Bias (DCB) is not a problem for the network solution because it can be absorbed by clock parameters. DCB will not affect the observation RMS of PPP solution, if the precise orbits and clocks used are estimated from data of the same receiver type. Therefore, DCB correction is only necessary for GPSPPP solution using IGS final products.

From Figure 5, RMS of PPP and network solutions are almost the same, namely 2 m for BDS and 1 m for GPS. This confirms that BDS range noise is about twice as large as that of GPS [4,8], although such a large range noise will not affect ZTD estimation if a proper weight is assigned to the range observations, it could cause problem for wide-lane ambiguity fixing using the Melbourne-Wübbena combination [28,29].

### 4.2. ZTD Assessment

**Figure 6.**
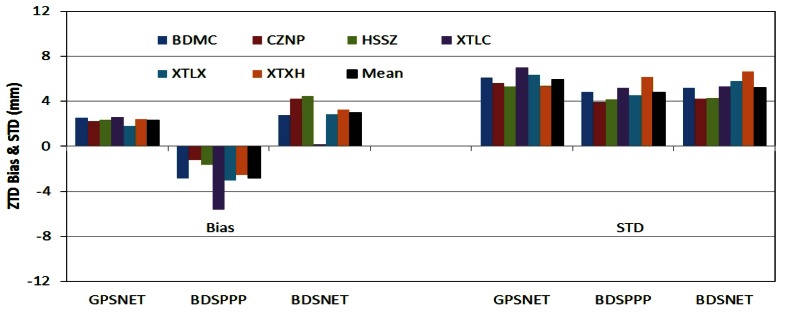
Bias (**left**) and STD (**right**) of ZTD derived at the six local stations from GPSNET, BDSPPP, and BDSNET compared with that derived from GPSPPP solution. The averaged Bias and STD are plotted in black at the end of each solution type.

Figure 6 shows the bias and STD of the ZTD from GPSNET, BDSPPP and BDSNET compared with that derived from GPSPPP for the six local stations. The bias and STD are also listed in Table 2.

From Table 2, the biases of the ZTD from GPSNET, BDSPPP and BDSNET with respect to that of GPSPPP solution are 2.3 mm, −2.8 mm and 3.0 mm; and the STDs are 5.9 mm, 5.8 mm and 5.2 mm, respectively. In generally, these differences are comparable to that of ZTD derived using different software packages [12]. From these statistics we can conclude that BDS can provide ZTD of the same quality as GPS does.

The bias between BDSPPP and BDSNET can be obtained by subtracting the bias of BDSPPP from that of BDSNET in Table 2. On average, this bias is about 6 mm, which is significantly larger than the bias between GPSPPP and GPSNET of 2.3 mm. One possible reason for such a bias might be the differences in stations coordinates estimated from BDS and GPS, especially in the vertical component [7] which is highly correlated with ZTD [30]. In our processing, the coordinates were fixed on those estimated from GPS data for all the four processing schemes. This bias could have a different effect on ZTD estimates in PPP and network mode and consequently result into such a large bias. We have tried to estimate station coordinates in PPP and the resulted ZTD has a smaller bias but a larger STD. Therefore, further investigations must be conducted with more data for overcoming such problem.

The disagreement, especially the bias, could be significantly reduced by applying accurate PCO and PCV corrections of the receiver antennas, and of course it will be further improved with more MEO satellites in operation.

**Figure 7.**
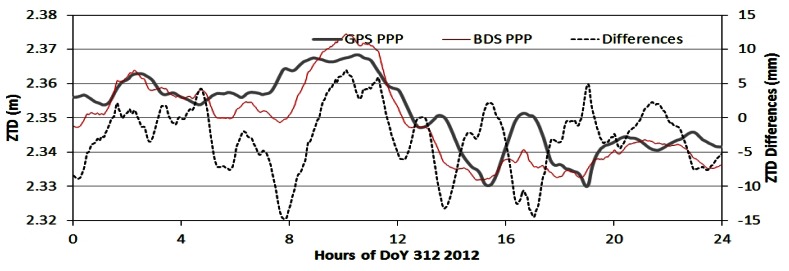
Comparison of ZTD derived using GPS and BDS data in PPP mode. The differences (BDSPPP-GPSPPP) are within +/−10 mm with a bias of −2 to −3 mm.

**Figure 8.**
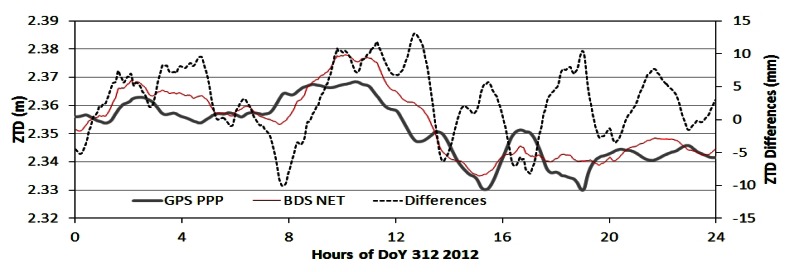
Comparison of ZTD derived from GPS data in PPP mode and from BDS data in network mode. The differences (BDSNET-GPSPPP) are within +/−10 mm with a bias of about 3 mm.

**Figure 9.**
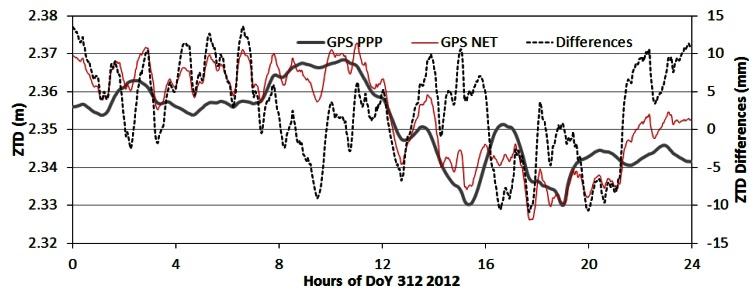
Comparison of ZTD derived from GPS data in PPP and network mode. The GPSNET ZTD has a larger variation than that of the GPSPPP. The differences (GPSNET-GPSPPP) are within +/−10 mm with a bias of about 2 to 3 mm.

As examples, Figures 7 to 9 show the ZTD time series and their differences of the four processing modes at station BDMC on the day 312 (November 7) in 2012. Figure 7 shows the ZTD time series of BDSPPP and GPSPPP and their differences. Their differences shown in black dash line are within +/−10 mm with a bias of −2 to −3 mm. Figure 8 ZTD of GPSPPP and BDSNET are plotted with their differences with a bias of about 3 mm.

Figure 9 illustrates ZTD derived from GPSPPP andGPSNET. The GPSNET ZTD has a larger variation than that of the GPSPPP. The differences (GPSNET-GPSPPP) are within +/−10 mm with a bias of about 2 to 3 mm.

## 5. Conclusions

The BDS regional system is now operational and providing positioning and navigation services over the Asia-Pacific region. Based on the studies on precise orbit determination and clock estimation, PPP and precise relative positioning, in this paper we have demonstrated its potential application in GNSS meteorology.

Data of a local network located close to Beijing with an inter-station distance of about 100 km is employed to assess the quality of BDS derived ZTDs. The BDS data is processed in PPP mode using precise orbits and clocks estimated from the BETS network. The data is also processed in network mode together with a few BETS stations far away in order to reduce their correlation between ZTD of the stations in a local network. The results are compared with the ZTD from GPSPPP solution using IGS final products.

The comparison shows that ZTDs derived from GPS and BDS agree with each other very well with a bias and STD of about 2 mm and 5 mm, respectively, which is similar to the ZTD differences derived with different software packages. The differences could be further reduced if more MEO satellites come into operation and precise PCO and PCV of receivers and satellites become available.

From such an assessment, we conclude that BDS can provide right now comparable ZTD products as GPS does. With its full constellation, BDS will significantly improve the performances of GNSS in meteorological study and weather forecasts.

## Figures and Tables

**Table 1.** Summary of observation models for ZTD estimation.

**Item**	**Models**
Observations	Undifferenced ionosphere-free code and phase combination of B1 and B2 with 30 s sampling
Elevation cutoff	7°
Weight for observations	Elevation dependent weight [E < 30°, 2*sin(E); otherwise 1]
Phase-windup effect	Applied
Earth rotation parameter	Fixed to IGS final ERP products
Tropospheric delay	Initial model + random-walk process (for details see text)
Ionospheric delay	Eliminated by ionosphere free combination
Satellite clock	White noise for network solution and Fixed for PPP
Receiver clock	White noise
Station displacement	Solid Earth tide, pole tide, ocean tide loading IERS Convention 2003
Satellite antenna PCO&PCV	Not corrected
Receiver antenna PCO& PCV	Not corrected

**Table 2.** Bias and STD of ZTD differences of GPSNET, BDSPPP and BDSNET with respect to that of GPSPPP.

**SITE**	**BIAS (mm)**			**STD (mm)**		
	
	GPSNET	BDSPPP	BDSNET	GPSNET	BDSPPP	BDSNET
**BDMC**	2.5	−2.8	2.8	6.1	4.8	5.2
**CZNP**	2.2	−1.2	4.2	5.6	3.9	4.2
**HSSZ**	2.3	−1.6	4.4	5.3	4.2	4.3
**XTLC**	2.6	−5.6	0.2	7.0	5.2	5.3
**XTLX**	1.8	−3.0	2.9	6.3	4.5	5.8
**XTXH**	2.4	−2.6	3.3	5.4	6.2	6.6

**Mean**	2.3	−2.8	3.0	5.9	4.8	5.2
